# Nrf2 Involvement in Chemical-Induced Skin Innate Immunity

**DOI:** 10.3389/fimmu.2019.01004

**Published:** 2019-05-07

**Authors:** Doumet Georges Helou, Stefan F. Martin, Marc Pallardy, Sylvie Chollet-Martin, Saadia Kerdine-Römer

**Affiliations:** ^1^Inflammation, Chimiokines et Immunopathologie, INSERM UMR996, University Paris-Sud, Université Paris-Saclay, Châtenay-Malabry, France; ^2^Allergy Research Group, Department of Dermatology, Faculty of Medicine, University of Freiburg, Freiburg, Germany; ^3^UF Auto-immunité et Hypersensibilités, Hôpital Bichat, APHP, Paris, France

**Keywords:** Nrf2, skin cells, innate immunity, contact sensitizers, xenoinflammation

## Abstract

Exposure to certain chemicals disturbs skin homeostasis. In particular, protein-reactive chemical contact sensitizers trigger an inflammatory immune response resulting in eczema and allergic contact dermatitis. Chemical sensitizers activate innate immune cells which orchestrate the skin immune response. This involves oxidative and inflammatory pathways. In parallel, the Nrf2/Keap1 pathway, a major ubiquitous regulator of cellular oxidative and electrophilic stress is activated in the different skin innate immune cells including epidermal Langerhans cells and dermal dendritic cells, but also in keratinocytes. In this context, Nrf2 shows a strong protective capacity through the downregulation of both the oxidative stress and inflammatory pathways. In this review we highlight the important role of Nrf2 in the control of the innate immune response of the skin to chemical sensitizers.

## Cutaneous Innate Immunity

Long ago, the skin was considered as a passive physical barrier protecting the internal organs from environmental assaults. In 1978, Streilein proposed that the skin has its own integrated immune function and introduced the term of skin-associated lymphoid tissues, “SALT.” He suggested that this specialized function is mediated by keratinocytes, Langerhans cells (LC) and immune-competent lymphocytes working in concert to ensure an efficient protection (1, 2). Nowadays, it is clear that the skin is an important part of the immune system. The main resident innate immune cells in the skin, such as LC in the epidermis or dendritic cell (DC) subpopulations, mast cells and macrophages in the dermis, supervise the most exposed organ, react rapidly to danger signals resulting from biological or chemical hazards and orchestrate the immune response. In addition, keratinocytes, and fibroblasts contribute to the immune response e.g., by chemokine release which leads to a rapid recruitment of circulating immune cells, mainly neutrophils and pro-inflammatory monocytes (3).

### Principal Resident and Recruited Innate Immune Cells

#### Keratinocytes

Although they form a complex and well-structured physical barrier constituting 90% of the epidermis, it is well established that keratinocytes play a major role in skin immunity, as a connector between environmental signals and the underlying immune cells (4, 5). In particular, keratinocytes can detect pathogens via their Toll-like receptors (TLRs) (6) and produce antimicrobial peptides (AMPs) such as cathelicidin and β-defensins (7). In addition, keratinocytes can produce several chemokines such as CCL1, CCL2, CCL5, CCL11, CCL13, CCL17, CCL18, CCL20, and CCL22 as well as pro-inflammatory cytokines such as IL-1β and IL-18 (8, 9). Interestingly, a recent study revealed that keratinocyte can produce IL-23 known to orchestrate chronic skin inflammation in psoriasis (10).

#### Langerhans Cells

Discovered by Paul Langerhans in 1868, LC are antigen presenting cells deriving from the monocytic lineage and represent 2 to 4% of the epidermis (11, 12). Not long ago, LC were defined as epidermal DC that migrate to the draining lymph node and prime antigen-specific T cells. Nowadays, LC are considered to be a subset of tissue-resident macrophages that share key properties with macrophages such as embryonic origin and their capacity to self-renew within the epidermis (13, 14). LC are characterized by their expression of langerin (CD207), a C-type lectin receptor (CLR) required for antigen recognition and internalization that result in the formation of Birbeck granules (15). While LC capacity to cross-present antigens to CD8^+^ T cells remains a topic of debate (16–18), it is proven that LC ensure immune tolerance and protection in contact hypersensitivity (CHS) through the inhibition allergen-specific CD8^+^ T cells and the activation of specific regulatory T cells expressing the inducible T cell co-stimulator (ICOS) (19).

#### Dendritic Cells

Skin DC are specialized cells that recognize and process antigens in the skin and then migrate to the draining lymph nodes to promote a T effector response. Skin DC present many subtypes such as dermal DC (dDC), conventional DC (cDC) and monocyte-derived DC (mDC) (20, 21). dDC represent the major DC population in the skin and could be divided into two populations: Langerin^+^ dDC that co-express the CD103 in mice or CD141^high^ in humans and Langerin^−^ dDC in mice or CD14^−^/CD1a^+^ in humans (22). Langerin ^+^ dDC and not LC play a key role in inducing immune responses and present a unique cross-presentation capacity among skin DC populations (18, 23, 24).

#### Macrophages and Monocytes

Macrophages are antigen-presenting cells better recognized for their innate functions, in particular their heterogeneity-defining phagocytic capacity during pathogen infection and tissue damage (25). Skin-resident macrophages have an embryonic origin and are self-maintained through proliferation, while infiltrating macrophages are derived from Ly-6C^hi^ inflammatory monocytes. Nevertheless, monocytes can enter the skin in the steady state and give rise to macrophages or DC, or re-enter the circulation without any differentiation (26). Resident macrophages can be classically activated (M1) through pattern-recognition receptors (PRR) signaling pathways triggering the production of pro-inflammatory cytokines such as IL-12, tumor necrosis factor (TNF) and IL-23. Moreover, macrophages can be alternatively activated during Th2-mediated immune responses under the influence of IL-4 and IL-13 (8, 27). These alternatively activated macrophages, referred to as regulatory M2 macrophages, display distinct functions and phenotypes according to their origin, but generally promote the resolution of skin inflammation partly via IL-10 and transforming growth factor-β (TGFβ) secretion (28, 29). In CHS, M2 macrophages secrete CXCL2 to attract dDC leading to an accumulation of DC–T cell clusters in skin. Therefore, depletion of monocytes/macrophages before elicitation suppresses the inflammatory response (30, 31).

#### Neutrophils

The rapid recruitment of neutrophils into the skin is a key protection mechanism against infections. In line with resident cell activation, in particular keratinocyte activation, neutrophils infiltrate the skin toward inflammatory mediators such as IL-1α, IL-1β, IL-6, TNF, CXCL1, and CXCL8 (8). Although neutrophils ensure an efficient protection against pathogens, their inappropriate activation can be responsible for severe skin inflammatory and autoimmune diseases (32). In psoriasis, neutrophils secrete IL-17 affecting thus the IL-23/IL-17 axis balance and maintaining the positive feedback loop of cutaneous inflammation (33, 34). While recent studies have evidenced that neutrophil recruitment to the skin during both phases of CHS is essential to induce a cutaneous inflammatory response, the debate is still open in relation to neutrophil involvement in antigen presentation (35–37).

### Pattern Recognition Signaling in Innate Immune Cells

Innate immune cells are the frontline of protection against invading pathogens and react immediately to infection or trauma. They are equipped with a large variety of PRR. These include TLRs, nucleotide binding oligomerization domain (NOD)-like receptors (NLRs) and RNA helicases (RIG-I (retinoid acid-inducible gene-I) that detect both microbial pathogen-associated molecular patterns (PAMPs) and endogenous damage-associated molecular patterns (DAMPs). In addition, scavenger receptors such as CD36 and macrophage receptor with collagenous structure (MARCO) are mainly expressed on macrophages and may recognize and internalize several self and microbes-derived ligands. PRR-PAMP/DAMP interactions induce the activation of a panel of intracellular signaling pathways including TLR- and NLR-dependent signaling pathways, kinases and transcription factors (38, 39). The downstream signal cascades are therefore associated with gene transcription and expression, leading mainly to the production of inflammatory cytokines. Among PRRs, TLRs represent the major class of receptors. They are highly expressed on sentinel cells including innate immune cells and non-immune cells like fibroblasts. TLRs are non-catalytic receptors formed by an extracellular binding domain and a cytoplasmic signaling Toll/interleukin-1 (IL-1) receptor homology (TIR) domain. However, some TLRs including TLR3, TLR7, TLR8, TLR9, TLR11, TLR12, and TLR13, are exclusively intracellular. They lodge in the endosomes and recognize principally nucleic acids. In parallel, TLR1, TLR2, TLR4, TLR5, TLR6, and TLR10 represent cell surface TLRs and recognize mainly structurally conserved motifs from pathogen membranes. TLR signaling is initiated with homo- or heterodimer formation following the recruitment of TIR domain-containing adaptors including MyD88, TRIF, TIRAP/MAL, or TRAM. MyD88 and TRIF are considered as the major downstream adaptors while TIRAP and TRAM are sorting adaptors that could mediate the signal from TLR to MyD88 and TRIF, respectively. The MyD88-dependent response is very common in different TLR subfamilies except TLR3, leading to the activation of nuclear factor-kappa B (NF-κB) and mitogen-activated protein kinases (MAPKs) and subsequently the expression of genes coding for key inflammatory cytokines. The TRIF-dependent pathway is mainly triggered by TLR3 and TLR4 and results in the production of interferon type I as well as NF-κB and MAPKs activation (40–42).

## Skin Exposure to Chemical Sensitizers

The skin is frequently exposed to chemical stress by organic chemicals or metal ions that can directly or indirectly challenge its immune components and may lead to T cell-mediated delayed type hypersensitivity reactions. As for pathogens, the recognition of these chemicals depends upon PRR expression on sentinel skin cells, mainly the innate resident immune cells. Therefore, these cells could define the global immune reaction: offensive or dangerous non-self, immune tolerance or harsh hypersensitivity.

### Allergic Contact Dermatitis

Allergic contact dermatitis (ACD) is a common inflammatory disease due to skin sensitization by chemical allergens. CHS is a well-accepted mouse model of ACD reproducing the multi-step process of skin sensitization and elicitation in human ACD. It is a manifestation of immune hypersensitivity, in contrast to irritant contact dermatitis (ICD) that results from a direct toxic effect of chemical agents on skin cells involving the innate immune system but not generating an adaptive immune response. The important prevalence of ACD, ~20%, is associated with an increased exposure to environmental and industrial products (43). Chemical sensitizers constitute an important group of low molecular weight contact allergens (MW < 500 Dalton) and behave as haptens. These include organic chemicals as usually found in fragrances, adhesives, preservatives, dyes and metal ions such as nickel, cobalt and chromium (44, 45). Complete haptens are highly reactive electrophilic molecules that bind directly to nucleophilic chains of self-proteins. Pro- and pre-haptens require enzymatic and oxidative transformation, respectively, to become reactive. Several factors drive protein haptenation within the skin proteome including the nucleophilicity, steric hindrance, competition for binding and local pH (46). While the nature of the hapten-modified proteins with functional relevance for ACD is largely unknown, it is clear that protein haptenation underlies the activation of innate immune and stress responses as well as the formation of T cell epitopes. In addition to haptenation, other types of protein modification may play an important role, e.g., oxidation as a consequence of reactive oxygen species (ROS) production induced by contact sensitizers. A disturbed redox balance may lead to thiol modification in proteins altering conformation and function (47). Thus, it has been shown that contact sensitizers induce the oxidation of cell surface thiols and that this contributes to the maturation of DC (48).

It is well established that following first skin penetration during the asymptomatic sensitization phase, haptens bind to skin proteins in order to become immunogenic. Haptens can induce oxidative stress responses in skin resident cells participating in a rapid recruitment of inflammatory cells, notably neutrophils, and an amplified activation of the cutaneous innate immunity. Subsequently, LC and dDC process haptenated proteins and migrate to the draining lymph node to prime hapten-specific T cells with a preferential Tc1/Th1 and Tc17/Th17 polarization. It is important to note here that this polarization could depend on hapten properties and the used experimental model (49). The elicitation phase of ACD results from repeated exposure to the same hapten and leads to skin infiltration by cytotoxic T cells. Local T cell activation by antigen-presenting cells is necessary for secreting interferon-gamma (IFN-γ) and IL-17, killing the haptenized cells and contributing to keratinocyte apoptosis (50). Hence, skin innate immune cell activation is maintained and amplified due to T cell cytokines and DAMPs. In this context, CD4+ and CD8+ T cells play an antagonistic role in the elicitation phase; while CD8+ T cells display cytotoxic functions, CD4+ T cells ensure a regulatory role to protect skin components from chronic inflammation. Furthermore, the resolution of inflammation relies on regulatory T cell recruitment to exert inhibitory functions in the inflamed skin. Indeed, many studies have reported a suppressive role of regulatory T cells, both during the sensitization and the elicitation phase of CHS, as their depletion results in a prolonged and exacerbated ear swelling response while their expansion reduces effector cells recruitment leading to a maintained suppression of CHS (51–53).

### Hapten Recognition by Skin Cells

The sensitization potential of haptens critically depends on their irritancy, considered as necessary to promote stress responses and to supply innate immune cells with further danger signals (44, 54). The direct hapten-induced inflammation, called xenoinflammation (55), produces DAMPs that are endogenous danger signals including hyaluronic acid (HA) fragments, biglycan, heat shock proteins, uric acid and extracellular adenosine triphosphate (ATP) (56–58). Some of these DAMPs trigger TLRs which normally recognize bacterial and viral components. This is the case for TLR2 and TLR4 in the CHS response. They are triggered by HA fragments or biglycan resulting in MAPKs and NF-κB activation and production of pro-inflammatory cytokines and chemokines. Among these cytokines are pro-IL1β and pro-IL18 which are important in contact dermatitis. Their cleavage to the mature bioactive forms is achieved by contact allergen-mediated activation of the pyrin domain containing 3 (NLRP3) inflammasome (59). This involves ROS and in some cases extracellular ATP. This TLR-NLRP3 inflammasome axis seems to be a common mechanism underlying skin inflammation as shown for several contact allergens in the CHS model and in human ACD.

Mice deficient in both TLR2 and TLR4 failed to develop CHS that was normal in germ-free mice. It was shown that contact allergens trigger the production of HA fragments, endogenous host-derived DAMPs which then trigger TLR2 and TLR4 (60). Moreover, a recent study has revealed that TLR3 signaling participates in the induction of chronic CHS (61).

Upon their penetration, chemical sensitizers induce ROS production in the skin, participating in the breakdown of the extracellular matrix (ECM) (56). Among the released endogenous signals, ROS and hyaluronidase-dependent HA degradation may lead to TLR2- and TLR4-mediated DC activation (56). Direct TLR activation has been shown for nickel and cobalt ions, known for their capacity to engage two non-conserved histidine residues in human TLR4, absent in mouse TLR4 (62, 63). Palladium works in a similar manner (64). For cadmium, a toxic heavy metal that can cause sensitization, an induction of ROS production has been shown and one recent study claims a role for TLR4 in the induction of mucin 8 in human airway epithelial cells via activation of MAPKs (65).

Along with TLRs, NLRs form cytosolic multi-protein complexes called inflammasomes that activate caspase-1 leading to the cleavage of the pro-inflammatory cytokines IL-1β and IL-18 into their bioactive forms (66). Different mouse models have supported the involvement of the inflammasome in skin innate responses to chemical sensitizers, and a role for the danger signal extracellular ATP in its activation via the P2X7 receptor was demonstrated. LC migration is also impaired in caspase-1^−/−^ mice exposed to the 1-fluoro-2,4-dinitrobenzene (DNFB) (67–70). Moreover, activation of the NLRP3 inflammasome by nickel in the human system has recently been reported (71). Many NLRs involved in the regulation of inflammation have been studied in the CHS model. It was shown that NLRP12^−/−^ mice display a reduced CHS response to either oxazolone or fluorescein isothiocyanate (FITC). This was associated with a reduced migration of DCs from skin to draining lymph nodes. Mice lacking NLRP10 also had reduced CHS to DNFB, but not to the irritant croton oil. Interestingly, the phenotype was also seen in mice lacking NLRP10 specifically in keratinocytes. In this study the reason for the reduced CHS response was not clear. A reduction of TNF-α and CXCL1 in skin was observed indicating a reduced skin inflammation and reduced neutrophil infiltration (68, 72).

Besides direct or indirect PRR triggering, proteomic-based approaches are nowadays suggesting that chemical sensitizers may trigger intracellular signaling pathways via direct interaction with cellular proteins. For example, FITC activates p38 and c-Jun N-terminal kinase (JNK) pathways through direct haptenation of specific amino acid residues within critical intracellular proteins in THP-1 cells (human leukemic monocyte) (73). Other molecules like DNFB, induce ROS production through direct interaction and depletion of cytoplasmic glutathione (GSH) in THP-1 cells (74).Within this context, we have previously used an omic-based approach to identify proteomic alterations in DCs upon exposure to sensitizers such as dinitrochlorobenzene (DNCB), cinnamaldehyde (CinA) and Nickel II sulfate. Spot pattern analyses pointed out a metabolic reprogramming in DC (oxidative stress responses and phase II metabolism) and evidenced a positive correlation between the reactivity and the potency of the applied chemical. A total of 100 proteins regulated by the sensitizers was identified and showed a specific profile for each sensitizer (75). Such an approach based on chemical reactivity provides thereby valuable biomarker candidates for sensitizers and would be of great benefit to immuno-toxicology discipline.

## Modulation of Skin Innate Immunity by Nrf2

As stated previously, redox homeostasis shapes the innate immune response to chemical sensitizers and protects against inflammation. Up to now, Nrf2 (nuclear factor erythroid-2-related factor 2) is considered as the master cytoprotective transcription factor that could dictate the outcome of oxidative and inflammatory pathways within innate immune cells. Although numerous studies have evidenced the regulatory role of Nrf2 in different inflammatory contexts, few have elucidated its capacity to manage skin primary immune responses to chemical sensitizers.

### Nrf2 in Oxidative/Xenobiotic Stress

Nrf2 constitute a major mechanism in the regulation of cellular oxidative stress, particularly the detoxification and excretion of both organic xenobiotics and toxic metals (76, 77). Given its cytoprotective functions, an evolutionary conservation of Nrf2 has been defined in vertebrates, with a protein homology ranging from 49% in zebrafish to 89% in cows when compared to humans (78). Nrf2 activity is tightly regulated by its cytosolic repressor Kelch-like ECH-associated protein 1 (Keap1). At basic state, Nrf2 is sequestrated in the cytoplasm by its inhibitor the Kelch-like ECH-associated protein 1 (Keap1). Keap1 forms an E3 ubiquitin ligase complex with Cullin 3 and Ring-box 1 and triggers polyubiquitination of Nrf2 by promoting its binding to Cullin 3. Ubiquitinated Nrf2 is then degraded by the 26S proteasome with a half-life of ~20 min. Exposure to oxidants or electrophilic stress cause conformational changes in the Keap1–E3 ubiquitin ligase complex that block Nrf2 ubiquitination and favor its accumulation. Nrf2 then rapidly translocates into the nucleus, dimerizes with the small Maf protein, and binds to the antioxidant-responsive elements (AREs) in gene promoters. Consequently, Nrf2 initiates the transcription of a battery of cytoprotective genes such as NAD(P)H: quinone oxidoreductase 1 (*nqo1*), heme oxygenase-1 (*ho-1*), glutathione S-transferase (*gst*), catalase, superoxide dismutase (*sod*) and glutamate-cysteine ligase modifier subunit (*gclm*) (76, 79, 80) (Table 1). Among Nrf2 downstream targets, *nqo1* is one of the most robustly inducible genes and its activation is strictly dependent on Nrf2 (81).

**Table 1:** Principal functions of five antioxidant enzymes encoded by Nrf2 target genes.

**Gene name**	**Abbreviation**	**Enzyme principal functions**
NAD(P)H quinone oxidoreductase-1	NQO1	Ensure reduction of quinone, scavenges superoxide
Heme oxygenase-1	HO-1	Catalyzes heme degradation to carbon monoxide
Catalase	CAT	Transforms hydrogen peroxide into water and oxygen
Superoxide dismutase	SOD	Catalyzes the dismutation of superoxide radicals to moleculer oxygen and hydrogen peroxide
Glutathione S-transferase	GST	Promotes the nucleophilic attack by glutathione on electrophilic molecules

Several Nrf2 activators have been identified, with a barn capacity to modify specific cysteines in Keap1 that is known to be a cysteine-rich protein with more than 25 cysteine residues. For instance, sulforaphane (SFN) is an isothiocyanate compound derived from cruciferous vegetables (such as broccoli) used as a pharmacological activator of Nrf2 (82). SFN has a preference to modify the cysteine 151 leading to an alteration in the Keap1-Nrf2-Culllin 3 complex conformation and to further stabilization and accumulation of Nrf2 (83).

### Nrf2 in Inflammation: Crosstalk With NF-κB and Inflammasomes

Experimental studies have evidenced that the absence of Nrf2 is always associated with an exacerbated inflammation in different animal model. The Nrf2 anti-inflammatory role is most likely attributed to a deep interaction between Nrf2 and many redox sensitive inflammatory pathways including NF-κB and inflammasomes.

**Figure 1:**
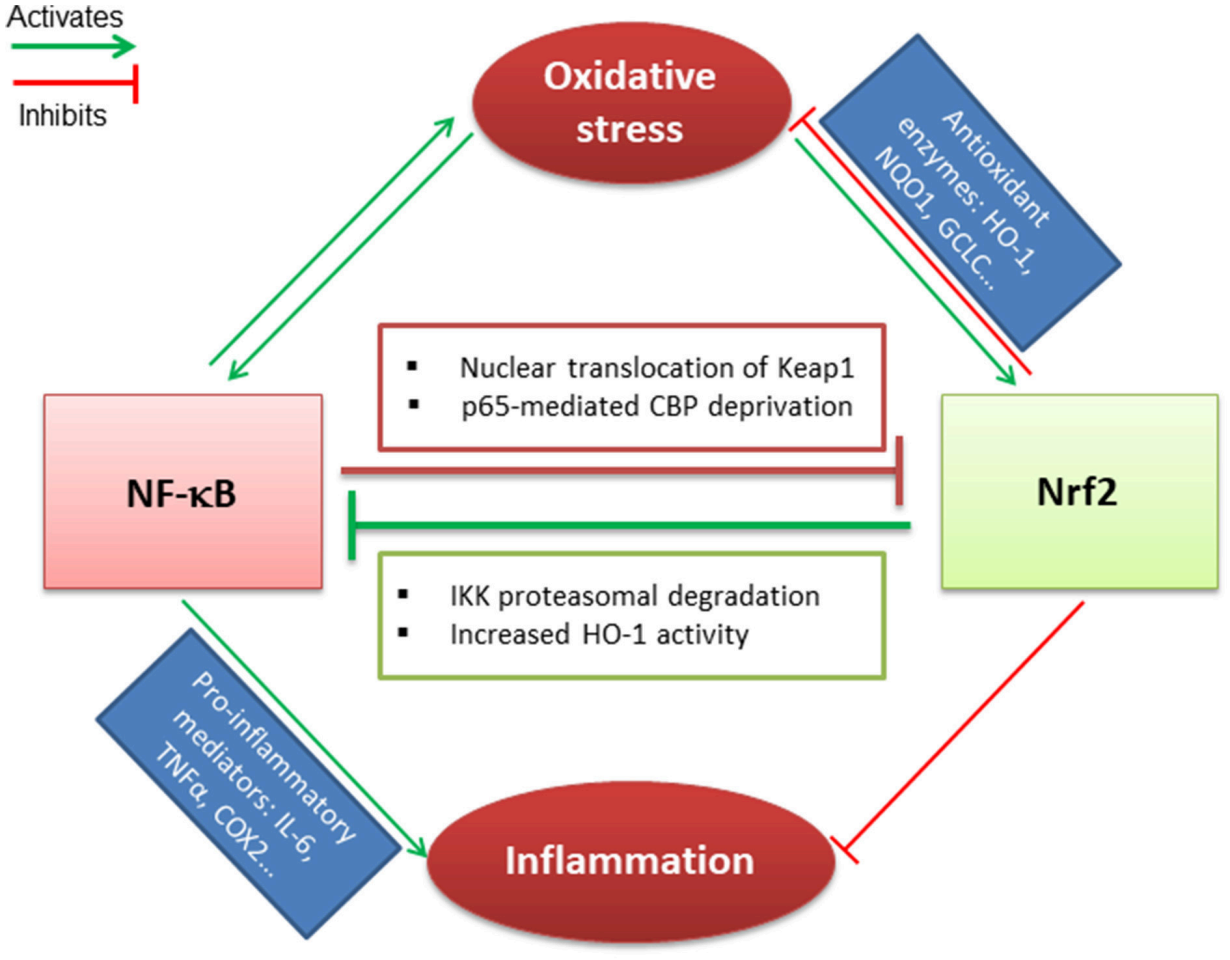
Crosstalk between Nrf2 and NF-κB transcription factors. Nrf2 and NF-κB related pathways are sensitive to oxidative stress that induces their activation. Nrf2 antagonizes NF-κB activation through IKK proteasomal activation and HO-1 activity end-products. In parallel, NF-κB downregulates Nrf2 via p65-mediated CBP deprivation as well as p65-induced Keap1 nuclear translocation. Nrf2 and NF-κB are regulated antagonistically: (i) if Nrf2 predominates, it decreases inflammation and oxidative stress through activation of antioxidant enzymes; (ii) when NF-κB predominates, it leads to pro-inflammatory mediators secretion and maintains the oxidative stress.

IκB, the negative regulator of NF-κB transcription factor, is phosphorylated by IκB kinase (IKK) during oxidative stress, leading to the release and nuclear translocation of NF-κB. The latter induces the transcription of pro-inflammatory mediators such as IL-6, TNF-α, and cyclooxygenase-2 (COX-2) (84). Nrf2 and NF-κB signaling pathways interact via multiple mechanisms. It has been demonstrated that Keap1 mediates IKK proteasomal degradation leading to NF-κB inhibition. This could result in a competitive binding between Nrf2 and IKK with Keap1 (85). The increased HO-1 activity that depends on Nrf2 activation, also participates in NF-κB inhibition (86). In parallel, the canonical NF-κB subunit p65 can alter the Nrf2 pathway. Indeed, overexpression of p65 leads to the translocation of Keap1 into the nucleus and the disruption of Nrf2-ARE interactions (87). In addition, p65 and Nrf2 compete for the transcriptional co-activator CBP (CREB-binding protein) that acetylates non-histone proteins to improve assembly of the transcriptional machinery (88) (Figure 1).

NF-κB activation is required for NLRP3 inflammasome priming, while Nrf2 activation could inhibit both pathways to limit inflammation. The activation of NLRP3 mediates IL-1β secretion followed by the process of cell death known as pyroptosis (89). Nrf2 reduces ROS levels through the activation of antioxidant target genes and inhibits NLRP3 priming. Indeed, a recent study has evidenced that Nrf2 is a key inhibitor of the NLRP3 inflammasome via the activation of the thioredoxin1 in cerebral ischemia reperfusion injury (90). Last, the multi-domain protein p62 provides a critical link between Nrf2, NF-κB and NLRP3 inflammasome pathways. Nrf2 and NF-κB contribute to p62 induction in oxidative stress, while a p62-mediated positive feedback loop supports NF-κB as well as Nrf2 activation through the autophagosomal degradation of Keap1. However, p62 inhibits the NRLP3 inflammasome (91, 92). Thus, p62 protein maintains a cellular homeostasis to ensure resistance to redox stress and inflammation depending on Nrf2.

Although literature screening suggests that Nrf2 participates in the control of inflammation through inhibitory crosstalk with redox sensitive pathways, emerging studies are suggesting a possible redox-independent regulation. Therefore, Nrf2 could directly regulate the expression of key genes to alleviate excessive immune reaction. We do not yet have enough hindsight to define all Nrf2 anti-inflammatory mechanisms; however there is a marked tendency to admit a greater role for Nrf2 in inflammation in relation with immune cell populations and pathophenotypes. In this regard, studies are continuing to report particular mechanism of regulation that is specific to chemical skin sensitization, notably to define cells- and/or chemical-specific mechanisms (86, 91).

### Regulation of Skin Innate Immunity by Nrf2

Nrf2 is a ubiquitously expressed transcription factor required for skin homeostasis mostly through modulation of the redox balance. Indeed, the electrophilic property of chemical sensitizers promotes the activation of Nrf2 in the skin. However, Nrf2 can modulate innate immune cell activation and function in different ways. Using a model of normal human epidermal keratinocytes (NHEK), it has been demonstrated that Nrf2 improves keratinocyte differentiation through the upregulation of specific differentiation markers such as loricrin and keratin 10 (93). Interestingly, a recent study has revealed that Nrf2 expression in keratinocytes ensures a rescue effect on UV-induced DNA damage and apoptosis in melanocytes (94). Furthermore, Nrf2 activation increases IL-36γ expression in keratinocytes leading to an increased proliferation (95). In psoriasis, Nrf2 is activated in response to inflammatory cytokines and enhances the expression of hyperproliferation-related keratins (96). Few studies have focused on the role of Nrf2 in the immune response of keratinocytes to chemical sensitizers, in particular its effect on cytokine production. Nevertheless, given its importance in sensing chemical stress, evaluation of Nrf2 activation in keratinocyte *in vitro* models becomes a useful tool participating in the prediction of chemical skin sensitization potential (97, 98). OECD guideline test 442D is based on a luciferase reporter of Nrf2 activation in the human keratinocyte cell line HaCaT (99).

Numerous studies have specifically investigated the role of Nrf2 in the DC response to chemical sensitizers in different cell models. We have demonstrated that chemical sensitizers such as nickel, DNCB and CinA were able to increase Nrf2 protein level in CD34-DC and THP-1 cells associated with an up-regulation of the target genes *ho-1* and *nqo1*, while irritants such as sodium dodecyl sulfate (SDS) and benzalkonium chloride (BZK) failed to induce Nrf2 accumulation. A positive correlation between Nrf2 target gene induction and chemical reactivity to cysteine has been established; however lysine-reactive chemicals were unable to activate Nrf2. These results proved that chemical reactivity of contact sensitizers provides necessary danger signals for Nrf2-dependent DC activation (100, 101). The use of Nrf2 knock-out (KO) mice allowed pinpointing the specific effects of Nrf2 in the DC response to chemical sensitizers. Indeed, using a proteomic approach, we identified a set of Nrf2-dependent and Nrf2-independent promising biomarkers that are regulated in hapten-induced DC activation. Thus, 7 ARE-containing Nrf2 targets were described relevant in response to chemical sensitizers: HSPA9, voltage-dependent anion-selective channel protein 1 (VDAC1), glutathione S-transferase omega-1 (GSTO1), ferritin light chain 1 (FTL1), peroxiredoxin 1 (PRDX1), SOD2 and transketolase TKT (75). Then using a transcriptional approach, we showed that the lack of Nrf2 in BMDC prevents the up-regulation of antioxidant genes in response to DNCB and CinA leading notably to a higher production of ROS and reduced levels of intracellular GSH. Furthermore, Nrf2 was able to control the chemically-induced apoptotic pathways in DC through the up-regulation of bcl-2 gene expression (102). Apart from chemical sensitizers, the majority of studies in different inflammatory contexts revealed that Nrf2 controls DC activation, notably the expression of co-stimulatory molecules such as CD86 and CD80 and affects the subsequent activation of T cells (103–106). Although Nrf2 may play a pivotal role in all skin immune cell populations, only its involvement in DC responses to chemical sensitizers was widely investigated, while its implication in other population such as LC, macrophages and neutrophils remains underappreciated. One question raises in all cases: is the anti-inflammatory role of Nrf2 independent from its capacity to regulate oxidative stress?

The best answer could be found in the study of Nrf2 implication in macrophage biology. A breakthrough study revealed that Nrf2 directly counteracts the transcription of pro-inflammatory cytokine genes revising a long-held view that Nrf2 regulates inflammation by redox control. This study provides a chromatin immunoprecipitation (Chip-seq)-based molecular approach suggesting that Nrf2 binds to the proximity of the IL-6 and IL-1β gene transcription start site, interfering thus with the recruitment of RNA polymerase. In contrast to the classically adopted hypothesis, this mechanism is totally independent from ROS levels and ARE, and is not a simple consequence to anti-oxidant upstream event (107). In line with these data, Nrf2 has the capacity to up-regulate the expression of MARCO and CD36, two scavenger receptors that are highly required for bacteria phagocytosis and elimination of apoptotic neutrophils, respectively, promoting thus the resolution of inflammation (108–111).

Although clear evidence about the role of Nrf2 in macrophage functions has emerged, its involvement in neutrophil biology remains a topic of debate. Many studies have argued a correlation between Nrf2 activation in neutrophils and the modulation of their hyper-reactive phenotype in inflammatory contexts such as sepsis and chronic periodontitis (112–114). More recently, a transgenic mouse model displaying Nrf2-specific deletion in myeloid cells was created. Interestingly, high Nrf2 mRNA levels were quantified in neutrophils of wound tissue and blood, 10-fold higher than in monocytes and lymphocytes. However, Nrf2 deletion in myeloid cells did not exacerbate the inflammatory response or alter the wound healing capability in this study (115). These striking results may put into question the role of Nrf2 in neutrophils suggesting the existence of redundant and alternative antioxidant mechanisms. Nevertheless, neutrophils are among the first cells to be recruited to chemically-treated skin in response to chemokines secreted by resident cells (36). Thus, their recruitment and their state of activation may indirectly rely on Nrf2 expression in skin resident cells.

**Figure 2:**
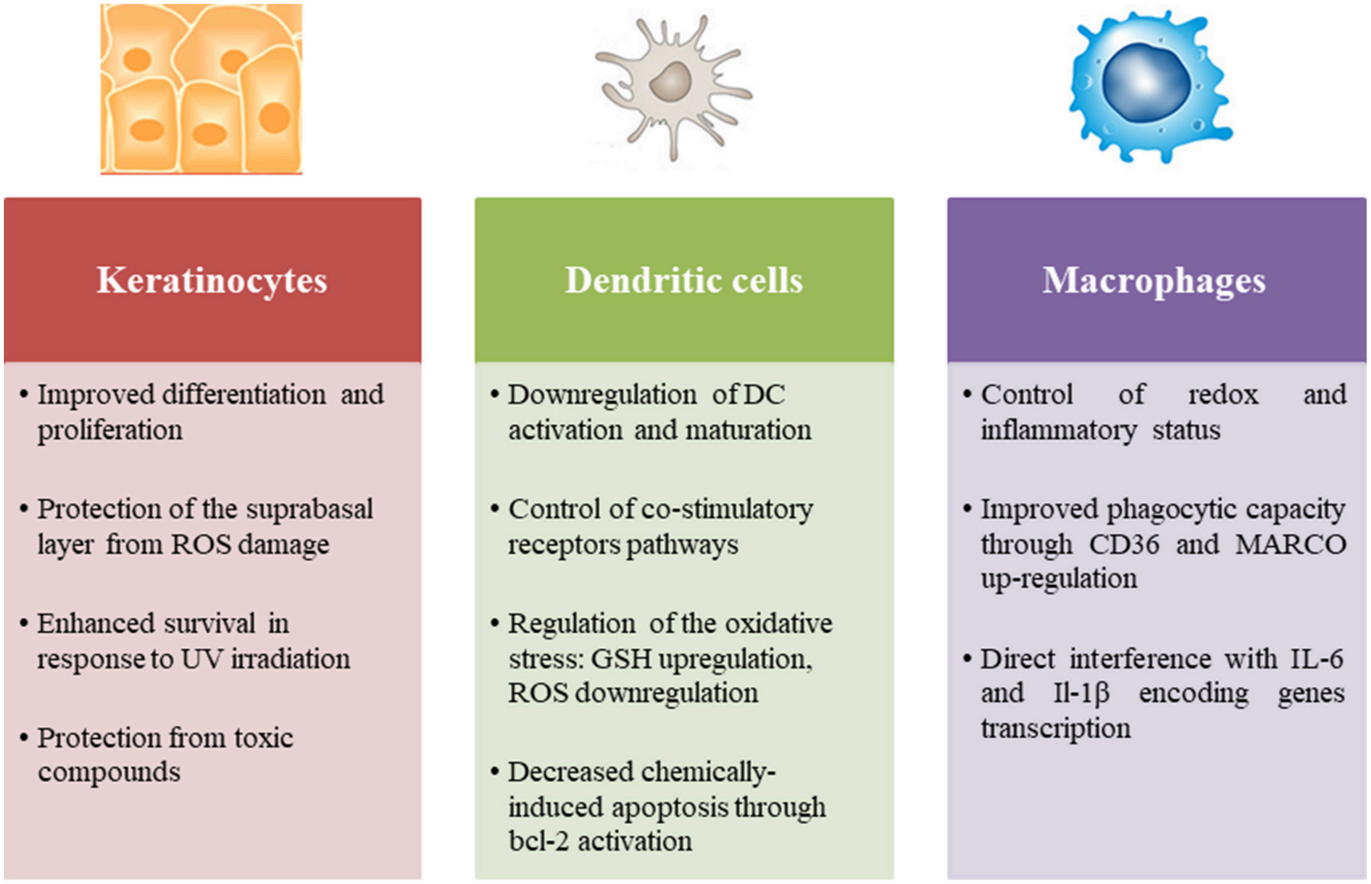
Role of Nrf2 in the principal skin innate immune cells including keratinocytes, dendritic cells, and macrophages.

**Figure 3:**
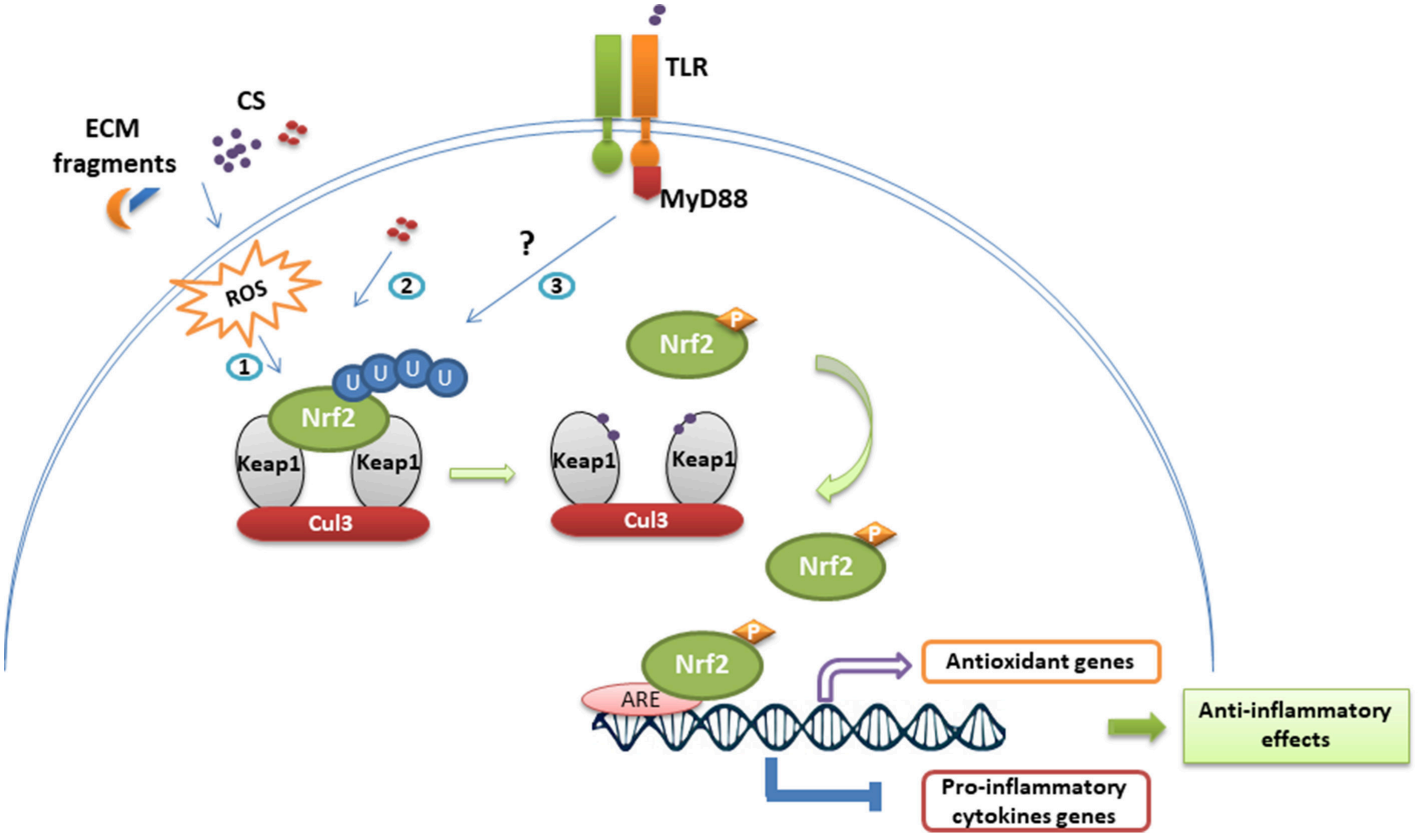
Nrf2 stabilization and anti-inflammatory effects following immune cell exposure to chemical sensitizers. In homeostatic conditions, Nrf2 is continuously repressed by the Keap1-Cul3 complex and ubiquitinylated. Exposure to chemical sensitizers (CS) activates the Nrf2/Keap1 pathway through different ways: (1) ROS-mediated activation resulting from CS irritancy and degradation of extracellular matrix (ECM), (2) direct chemical modification of Keap1, (3) TLR-dependent activation. Thus, modifications on Keap1 lead to Nrf2 stabilization and phosphorylation. Nrf2 translocates then to the nucleus where it binds to ARE sequences promoting the transcription of antioxidant genes and inhibiting the transcriptional activity of several genes encoding pro-inflammatory cytokines.

Altogether, Nrf2 is a key regulator of innate immune cells, in particular the most common pathway induced by chemical sensitizers (Figures 2, 3). Consistently, Nrf2 is actually considered as a key biomarker able to differentiate contact sensitizers from non-sensitizers. Furthermore, the important need for alternatives to animal testing, led to the development of a keratinocyte-based *in vitro* assay, relying on Nrf2 activation as a key event to identify sensitizers (97, 99, 116). Although numerous toxicological studies have characterized at the molecular level the underlying mechanisms in the principal skin cells (keratinocytes and DC), few immunological studies took into consideration the complexity of interactions between the different actors of the immune system that could drive the response to chemical sensitizers in an unpredictable way. To overcome the lack, our group addressed the question *in vivo*, using a Nrf2 KO mouse model. In response to moderate and strong chemical sensitizers, CinA and DNCB respectively, Nrf2 decreases skin inflammation during the elicitation phase of CHS in a concentration-dependent manner. Indeed, the mouse ear swelling test showed an approximatively 2-fold higher percentage of ear thickness increase, 48 h after challenge. Moreover, using the local lymph node assay (LLNA), we detected an enhanced proliferation of lymph node cells during the sensitization phase of CHS, suggesting that Nrf2 controls activation and migration of skin DC. This enhanced proliferation was not observed exclusively in response to DNCB and CinA that react specifically with cysteine residues, but also in response to trimethylaluminium (TMA) that reacts with lysine residues and to mixed reactive compound like isophorone diisocyanate (IPDI). Interestingly, Nrf2 is also able to regulate skin irritation in response to croton oil which fails to induce Nrf2 *in vitro* (117). This last result is in accordance with a recent study showing the Nrf2 target gene *nqo1* controls skin irritation in response to croton oil through the protection and maintenance of dendritic epidermal T cells (DETC) (118).

These *in vivo* studies in the CHS model clearly show that the threshold for sensitization to contact allergens is significantly lowered in Nrf2 KO mice. This is most likely due to the increased inflammatory response in absence of Nrf2 due to loss of redox homeostasis. A single contact sensitizer application, but also croton oil application produces a significantly increased ear swelling compared to wildtype mice. The fact that Nrf2 KO mice can even mount CHS responses to weak contact sensitizers which fail to do so in wildtype mice underlines the crucial role of the Keap1/Nrf2 system in balancing inflammation and immuno-regulation which are inversely correlated. A recent *in vivo* study conducted by our group has dissected the mechanisms allowing Nrf2 to regulate CHS, more specifically the early immune events following skin sensitization. Interestingly, Nrf2 inhibits neutrophil recruitment to the skin that was dependent on ROS elimination and is able to shorten the persistence of neutrophils in the inflamed skin. Indeed, Nrf2 enhances neutrophil clearance by resident macrophages through a direct activation of the macrophage-specific gene encoding CD36, which is essential for efferocytosis. In this regard, the mentioned study reports a novel Nrf2-regulated mechanism of CHS development independently from the redox balance (119). Of note, the sum of different mechanisms allows Nrf2 to play a remarkable role in inhibiting the sensitization process.

Interestingly, we have evidence now that Nrf2 downregulates the adaptive immune response in CHS indirectly via the modulation of the early innate immune response to chemical sensitizers in xenoinflammation. Thus, Nrf2-dependent immuno-regulation started very early after chemical sensitizer diffusion into the skin, highlighting the necessity of an optimal activation of Nrf2 during the first contact between the chemical and the skin in order to avoid the set-up of the sensitization process. In particular, variability in the level of Nrf2 activation in response to chemical sensitizers could bring important elements explaining the particular susceptibility of certain individuals to develop cutaneous allergies. The same concept applies to define a polymorphism in allergic patients based on the level of Nrf2 activation in the skin. Indeed, we can suggest that patients with ACD associated with low expression or reduced inducibility of Nrf2 in skin cells may benefit from specific treatment based on skin Nrf2 activation. However, a therapeutic strategy dependent on Nrf2 in the ACD should take into consideration the benefit/risk ratio. In particular, prolonged activation of Nrf2 can be dangerous and lead to alteration of the epidermal barrier, uncontrolled hyper-proliferation of keratinocytes and pro-tumorigenic effects related to immunosuppression.

## Future Directions

Taken together, Nrf2 is a major regulator in skin immune responses to chemical sensitizers. These latter trigger inflammatory responses in skin innate immune cells associated with Nrf2 activation. Consequently, a battery of cytoprotective enzymes counterbalances the cellular oxidative stress mainly through ROS elimination. Although Nrf2 regulates pro-inflammatory cytokine pathways and efferocytosis in macrophages via direct gene regulation, further studies are required to better understand the mechanisms of Nrf2 contribution in other skin innate immune cells. Nowadays, there is a growing interest in new pertinent tests to evaluate the sensitization potential of frequently used chemicals that are flooding the market every day, with new potential sensitizers. On the other hand, several Nrf2 activators, such as modified triterpenoids, are proving anti-inflammatory protective effects in clinical trials (120, 121). Thus, the importance of Nrf2 emerges not only in risk assessment but also as a potential therapeutic target in chemically-induced skin inflammation and other diseases such as cancer.

## Author Contributions

DH wrote the manuscript and designed figures. MP critically reviewed the manuscript. SM, SC-M and SK-R critically reviewed the manuscript and applied important modifications. All authors have approved the last version.

### Conflict of Interest Statement

The authors declare that the research was conducted in the absence of any commercial or financial relationships that could be construed as a potential conflict of interest.

## Abbreviations

ACD: Allergic contact dermatitis
ARE: Antioxidant response element
ATP: Adenosine triphosphate
CHS: Contact hypersensitivity
CinA: Cinnamaldehyde
DAMP: Damage-associated molecular patterns
DC: Dendritic cells
DNCB: dinitrochlorobenzene
GSH: Glutathione
HO-1: Heme oxygenase-1
IKK: IκB kinase
Keap-1: Kelch-like ECH-associated protein 1
KO: Knock-out
LC: Langerhans cells
NF-κB: nuclear factor-kappa B
NLR: (NOD)-like receptors
NQO1, NAD(P)H: quinone oxidoreductase 1
Nrf2: Nuclear factor erythroid-2-related factor 2
PAMP: Pathogen-associated molecular patterns
PRR: Pattern-recognition receptors
ROS: Reactive oxygen species
Tc: Cytotoxic T cells
Th: Helper T cells
TLR: Toll-like receptors
TNF: Tumor necrosis factor.
